# Review of "The Physical Measurement of Bone", Edited by C.M. Langton and C.F. Njeh

**DOI:** 10.1186/1475-925X-4-37

**Published:** 2005-06-14

**Authors:** Amit Gefen

**Affiliations:** 1 Department of Biomedical Engineering, Faculty of Engineering, Tel Aviv University, Tel Aviv 69978, Israel

This book is intended for biologists, medical physicists, biomechanical engineers and clinicians who are involved in bone studies. For these practitioners, the book is a comprehensive review of physical measurement techniques applied to bone, with useful emphasis on practical aspects such as protocols of measurement, sample preparation and sources of errors. Chapters were written by leading scientists in the relevant fields, and overall, the outcome is a well-written, comprehensive and useful textbook that will definitely serve the biomedical community.

The book contains 18 chapters classified into four major sections: Introduction, Invasive Techniques, Ionizing Radiation Techniques, and Non-Ionizing Techniques. As typically occurs with books that are collections of chapters from several authors, the quality is not uniform throughout. For example, while some figures are clear and illustrative, other figures suffer poor quality or were reproduced at a too low resolution. The important points of strength and weakness are summarized below.

The introduction includes a comprehensive review of bone anatomy and physiology, in which each skeletal component – bone cells, matrix and mineral – is separately addressed. It would have been useful here to also refer to tissues that connect to bone (tendons, ligaments, cartilage) and produce mechanical loads which act on bone (muscles) and particularly, to the interfaces between bone and these soft tissues. A review of the pathophysiology, etiology, epidemiology, and economic impact of osteoporosis follows, but other, less common bone diseases are only briefly mentioned. A useful chapter on biology safety considerations, with focus on bone studies, is also included in the introduction. I found this chapter particularly helpful as a guide for students or laboratory technicians who become involved in preparing bone sections for histopathology or mechanical testing. Typically, the information on how to avoid accidents such as when dealing with bone saws, chemicals for demineralizing, fixing and staining bone samples, radiation, and biological waste, is posted in internal laboratory or institutional regulations. This is the first textbook chapter that integrates this information specifically for experiments with bone, and I will certainly make it an obligatory reading material for those involved in bone studies in my laboratory. The last chapter in the introduction concerns instrument evaluation. While appreciating the important information provided on measurement errors, precision, and accuracy, I found this chapter to be too general in nature, and was not convinced that it should have been included in a textbook that focuses on bone measurements. For example, the classical Bland and Altman paper in Lancet (1986) [1] which is discussed in detail as related to bone mineral density measurements, originally referred to flow and oxygen saturation measurements, and can be generalized for bone as for any other application. Accordingly, this chapter could have fit a textbook on biomedical instrumentation, but it does not contain contents that are specific enough for bone studies.

The second section, on invasive techniques, puts heavy emphasis on the chapter which concerns mechanical testing of bone. Unfortunately, the contents of this chapter substantially overlap information already provided in the Bone Mechanics Handbook [2] which is a classic in this field. I was disappointed to find out that the tabulated data of mechanical properties for cancellous bone, cortical bone and individual trabeculae mostly summarize publications from the 1970's and 1980's (with few references to publications from the 1990's). Publications from 2000's (e.g. [3,4]) are not covered. I would have expected this new book to cover a more recent literature than that covered in the Bone Mechanics Handbook published on 2001 [2] or in Prof. J.D. Currey's book "Bones: structure and mechanics" published on 2002 [5], particularly because of the growth in attention to bone mechanical properties as osteoporosis becomes a major epidemic. The issue of bone viscoelasticity, which is discussed in detail in [2], is only briefly mentioned here in terms of the dependence of the elastic modulus of cortical bone on the strain rate. On the contrary, I found the subsequent chapters on bone histomorphometry, microscopy and related techniques very useful, specifically because these chapters include detailed procedures for bone histological staining as well as for immunohistochemistry, immunofluorescence, confocal microscopy and scanning electron microscopy analyses.

The third section concerns ionizing radiation techniques and specifically, absorptiometric methods to asses bone mineral density such as DXA, quantitative CT, peripheral QCT and micro-CT. Again, the parts which describe the fundamentals of radiation physics, interactions of x-rays with matter and radiological instrumentation may be too general for this type of book, but the applications to assessment of bone quality in humans and animal models are useful. A remarkable drawback in this section is that nearly all attention is given to bone mineral density, and very little information is provided on morphological parameters of bone that are demonstrated by the CT-based imaging modalities. Specifically, measurements of trabecular separation, trabecular thickness, bone volume fraction, trabecular number, trabecular connectivity etc., and accuracy of these measurements using clinical QCT and micro-CT are not addressed. Relations between these morphologic parameters, which are important characteristics of cancellous bone, are also not mentioned. Although the chapter on MRI that is included in the fourth section (non-ionizing techniques) partially concerns these issues, the reader is lacking information on morphological parameters that can be measured specifically by CT-based methods, and on the relation of accuracy to CT resolution for each morphological parameter.

The last section is dedicated to non-ionizing techniques for bone studies. These include imaging methods such as MRI and ultrasound, as well as finite element and animal models. The chapter on ultrasound measurements of bone is particularly worth mentioning as a well-written review on the technological aspects, accuracy and precision and clinical applications. The chapter on finite element analysis covers up-to-date modeling approaches based on CT cross-sectional images at the whole bone level, or based on micro-CT or micro-MR images at the trabecular level. Geometrical model reconstructions from cross-sectional images and bone mechanical properties relevant to structural modeling are described in adequate detail, but meshing techniques and the effect of mesh density and element selection on the predicted stresses and deformations in bone were not addressed (qualitatively or quantitatively). This may disappoint a reader who was hoping to use this chapter as a practical, rather than a theoretical guide for model development. A second drawback relates to the discussion on models at the trabecular levels, where generic models of the trabecular architecture are ignored. Generic modeling of trabecular bone, which utilizes the theory of cellular solid mechanics, is a complementary modeling approach to specimen-specific (micro-CT or micro-MR image based) modeling. Generic models allow to study the mechanics of 'typical' trabecular bone samples [6,7], as opposed to specimen-specific models which may include abnormalities or anatomical variations that are specific to the studied specimens [7]. The concluding chapter in the last section concerns animal models to study bone, and particularly, osteoporosis. I found this chapter to be extremely useful for experimentalists who weigh alternative models for an experimental design of an animal study of osteoporosis.

Having considered the points of strength and weakness in the different sections of this book as discussed above, I certainly recommend individual researchers and laboratories who deal with bone studies to hold a copy of this book. It should be noted that for those investigators focused on biomechanics of bone, it is not a substitute for the classics "Bone Mechanics Handbook" [2] and "Bones" [5], but rather, a complimentary book with particular added value in biology-oriented topics of safety considerations, microscopical techniques and animal model specifications. Bioengineering students, medical physics students and perhaps biology students at the graduate levels will also find this book useful for obtaining insight into the broad field of bone analysis.

## Abbreviations

DXA = dual x-ray absorptiometry

CT = computed tomography

QCT = quantitative computed tomography

MRI = magnetic resonance imaging

MR = magnetic resonance
